# Association between adult attachment and mental health states among health care workers: the mediating role of social support

**DOI:** 10.3389/fpsyg.2024.1330581

**Published:** 2024-03-07

**Authors:** Yahui Yang, Kaichao Chen, Kaiwen Liang, Wanyi Du, Jiamei Guo, Lian Du

**Affiliations:** ^1^Department of Psychiatry, The First Affiliated Hospital of Chongqing Medical University, Chongqing, China; ^2^Health Management Center, The First Affiliated Hospital of Chongqing Medical University, Chongqing, China

**Keywords:** mental health, healthcare workers, medical student, mediation analysis, adult attachment style, social support

## Abstract

**Background:**

To determine the relationships between attachment style, social support, and mental health states, as well as the mediation mechanism within this relationship, we conducted a survey among healthcare workers during the coronavirus disease 2019 (COVID-19) epidemic quarantine.

**Methods:**

The survey assessed their mental health states, adult attachment style, social support, and some other relevant information. Mental health states were represented by the overall state of sleep, physical and emotional assessment. A multiple mediator model was used to explain how social support could mediate the relationship between attachment and mental health states during COVID-19 quarantine.

**Results:**

Our findings revealed that 33.3% of the participants experienced emotional issues, 8.5% had sleep problems, and 24.9% reported physical discomfort. The direct effect of adult attachment styles on mental health states during COVID-19 quarantine was significant (c′ = −0.3172; *p* < 0.01). The total indirect effect also showed statistical significance (ab = −0.1857; *p* < 0.01). Moreover, the total effect of adult attachment styles on mental health states was −0.5029 (c = −0.5029; *p* < 0.01). Subjective social support and utilization of social support play mediating roles in the relationship between attachment style and mental health states, respectively (ab_1_ = −0.1287, 95% CI: −0.9120 to −0.3341, ab_2_ = 0.0570, 95% CI: −0.4635 to −0.1132).

**Conclusion:**

These findings highlight social support played a mediation role between attachment style and mental health states. Thus, offering social support during a crisis might be useful for those individuals with an insecure attachment.

## 1 Introduction

Stress refers to the state of physical and mental tension that individuals experience during the process of adaptation (Khansari and Murgo, 1990), and it could increase the susceptibility to psychological problems such as insomnia, physical discomfort and negative emotions (Pickering, 2001; Gardani et al., 2022; Wolkenstein et al., 2022). During the quarantine for coronavirus disease 2019 (COVID-19), people’s daily life and work were inconvenienced, and their physical and mental health were severely impacted (Levy, 2022; Cheng et al., 2023). Previous studies have pointed that the prevalence of depression and post-traumatic stress disorder among those who have been quarantined is higher than among those who have not been quarantined (Ripon et al., 2020). Furthermore, medical staff who have been quarantined may experience severe symptoms of post-traumatic stress (Rodríguez and Sánchez, 2020). It is evident that, when confronted with the same epidemic quarantine pressure, healthcare workers experience heightened psychological stress, however, an individual’s mental health state after suffering from stress can be quite different. Not everyone exposed to similar stressful life events will develop negative outcomes or a loss of functioning (Keller et al., 2012; Schönfeld et al., 2017).

Attachment theory was formulated in the 1960s by Bowlby (1969) and Li et al. (2023). This theory proposes that early attachment is formed through interactions with primary caregivers during infancy (Bowlby, 1980). The interactions between infant and caregiver are internalized into “internal working models” that usually continue into adulthood (Shemmings, 2006). These schemas of social situations provide a template for interpersonal behavior, known as the adult attachment orientation (Gittleman et al., 1998). Berman and Sperling (1994) defined adult attachment as an individual’s stable tendency to seek and maintain an attachment figure, which can provide both physical and psychological security. This stable tendency is regulated by the internal working model of attachment. The internal working model of attachment can regulate and manage stressful events (Bowlby, 1982; Shaver and Mikulincer, 2007), promoting adaptive responses to threats. Bartholomew (1990) also categorized adult attachment into four types, namely secure, preoccupied, dismissing, and fearful. Adult attachment may play a crucial role in determining the level of distress individuals experience in stressful events (Pietromonaco et al., 2013; Zhang et al., 2022). Research has found that secure attachment is believed to enhance individuals’ coping skills, personal worth, and self-efficacy, thereby reducing anxiety, promoting positive and constructive strategies for dealing with environmental stressors, and improving emotional regulation (Ogle et al., 2015). Insecure attachment makes individuals more susceptible to the effects of stress, increasing the risk of developing adverse mental health symptoms (Bryant, 2016).

Social support is another key factor that could have a protective effect on mental health. Social support is typically defined as the assistance provided by others, usually including family members, friends, colleagues, or other significant individuals (Saltzman et al., 2020). Previous research has shown that positive social support can not only improve one’s sense of self, but also buffer the negative impacts of stressful events (Li et al., 2021). Perceived social support plays a protective role in the development of post-traumatic stress disorder, helping to alleviate the severity of symptoms. Less perceived social support has been shown to be related to fewer use of adaptive coping strategies (Johnstone and Feeney, 2015). Individuals with insecure attachment traits often have difficulty finding supportive figures and may feel less satisfied in social interactions (Collins and Feeney, 2004; Reblin and Uchino, 2008; Danielsen et al., 2009). In conclusion, attachment styles influence the individuals’ ability to get and feel social support. Thus, we propose that social support could play an important role in the effect of adult attachment on mental health status after being exposed to stress.

**FIGURE 1:**
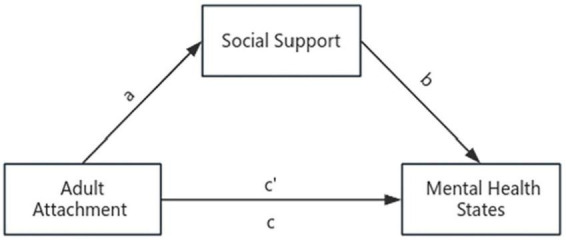
Attachment affects people’s perception of social support, which in turn affects their corresponding response states. a: the effect of adult attachment patterns on social support. b: the effect of social support on mental health states after controlling for the influence of adult attachment patterns. c: the total effect of adult attachment patterns on mental health states. c’: the direct effect of adult attachment on mental health states.

Therefore, we chose quarantine during COVID-19 as a stressful event, aiming to understand why people have different mental health states after this similar stressful event. Up to now, only a few studies have examined attachment in individuals during the COVID-19 pandemic (Liang et al., 2021; Costa-Cordella et al., 2022). Previous studies have shown that secure attachment appears to be a protective factor for mental health during the COVID-19 pandemic (Liang et al., 2021; Costa-Cordella et al., 2022). However, the existing studies have predominantly centered on the general population (Liang et al., 2021; Costa-Cordella et al., 2022), with a noticeable dearth of research focusing on healthcare workers. Furthermore, there is little evidence as to the mediating role of social support in the association between attachment and mental health state in healthcare workers. Thus, we assessed the physical sensations, sleep and emotions of medical staff and medical students during quarantine, and integrated these three aspects to represent the level of individual mental health state. In addition, we utilized the mediation analysis to evaluate the internal mechanisms of the impact of adult attachment on the mental health states. We hypothesized that: (1) secure adult attachment is positively correlated with the mental health status of healthcare workers; hypothesis (2): social support mediates the relationship between attachment style and mental health states; the hypothetical model is shown in Figure 1.

## 2 Materials and methods

### 2.1 Participants

This study was a cross-sectional study. The survey data was collected using the WeChat-embedded “Questionnaire Star” software program,^1^ which is widely used in survey research. Study invitation and data collection forms were linked to a Quick Response code (QR code) that was distributed to various official WeChat work groups of the First Affiliated Hospital of Chongqing Medical University (CMUS), including groups of doctors, nurses, clinical department staff, and medical students. In those WeChat groups, we described a clear explanation of the survey’s purpose and emphasized that participation was entirely voluntary. On the first page of the questionnaire, we also provided an introduction to the survey, including the purpose, the research team, and the anonymity and confidentiality of the participants’ information. Then, the respondents were required to click a button on the bottom of the first page indicating their agreement with this survey. To prevent duplicate responses, the survey was programmed to ensure that participants could only participate once. This study received approval from the Ethic Committee of the First Affiliated Hospital of CMUS. A total of 1,053 medical staff completed the questionnaire, but 6 responses were excluded due to insufficient response time or consistent response styles. Ultimately, 1,047 valid questionnaires were obtained, with an average age of 31.47 ± 12.503 years, ranging from 17 to 75 years, resulting in an effective rate of 99.43%.

### 2.2 Procedures

The survey questionnaire was formed with the help of the Department of Psychiatry and Health Management Center at the First Affiliated Hospital of CMUS. First, we developed an original version of the questionnaire and requested suggestions from specialists in the psychiatric field. Subsequently, we adjusted the questionnaire based on feedback from a small group of medical workers, incorporating their suggestions to refine the wording and style. The questionnaire aimed to collect information about the dependent variable (mental health states), key independent variables related to adult attachment styles, level of social support, and demographic characteristic.

### 2.3 Instruments

#### 2.3.1 Socio-demographic information

A sociodemographic data questionnaire was designed by the researchers, which collected information on age, gender, and education (junior high school or less/senior high school/college or more), respectively.

#### 2.3.2 Mental health states

Mental health states were assessed using a 10-point VAS to assess and monitor specific factors, with a scoring range from 0 (none) to 10 (highest level) (Hull et al., 2020). VAS were developed for a wide range of research and clinical applications, including mood, suicidal intent, depression, anxiety, dyspnea, craving for cigarettes, quality of sleep, functional abilities, acute pain, chronic pain, nausea, grip, disability, and vigor (McCormack et al., 1988; Wewers and Lowe, 1990). The VAS became used as a measure of health-related quality of life from the 1970s, following Priestman and Baum’s (1976) study of cancer patients. This study used VAS scores to record mood state (from best to worst), physical health state (from best to worst) and sleep state (from best to worst). Higher scores indicated poorer health states. The summation of mood state, physical health state, and sleep state scores were considered the current mental health states (mental health states).

#### 2.3.3 Adult attachment style

Adult attachment style was assessed using the revised version of the Adult Attachment Scale, revised by Collins (1996), which has demonstrated good reliability and validity in previous studies conducted in China (Wu et al., 2004). It consists of 18 items scored on a 5-point scale ranging from 1 (Not at all characteristic of me) to 5 (Extremely characteristic of me), organized in three subscales: anxiety, comfort with closeness, and comfort with depending on others. According to Brennan et al. (1988) these subscales are further organized in two dimensions: attachment anxiety and attachment avoidance. Individuals who score highly on the attachment anxiety tend to display an excessive concern with their own distress and negative emotions and to overreact to their negative feelings in order to elicit support from others. Individuals who score highly on attachment avoidance tend to seek distance cognitive and behavioral) from the stressful event, seeming less sensitive to it, and avoid seeking emotional or instrumental support from others (Mikulincer and Florian, 1995; Lopez and Brennan, 2000). Higher scores are indicative of more anxious and/or avoidant working models (i.e., insecure working models). In this sample, the reliability values were 0.71 (Avoidance) and 0.88 (Anxiety). For comparison analyses of the attachment profiles, the participants were assigned to their respective attachment styles (secure, preoccupied, dismissing, and fearful) based on whether their scores on the attachment-related anxiety and avoidance dimensions were above or below the scale midpoint (3). In this study, preoccupied, dismissive, and fearful styles were all categorized as insecure attachment styles.

#### 2.3.4 Social support

Social support was assessed using the version of the Social Support Rating Scale (SSRS) compiled by Xiao (1994), which is widely used in China because of its strong reliability and validity. The scale consists of 10 items and is divided into three dimensions: objective support, subjective support, and utilization of support. The overall perception of social support is determined by the total scores obtained in each dimension. In this study, the internal consistency of SSRS was 0.78 by Cronbach’s alpha.

### 2.4 Data analysis

In this study, all analyses were performed using the Statistical Package for the Social Sciences (SPSS 26.0, IBM). First, descriptive analyses were conducted to examine the demographic characteristics, mental health states and the adult attachment. Second, a Pearson correlation analysis was used to explore the relationships between mental health states, social support, and attachment styles. Last, a mediation analysis was conducted using model 4 of the PROCESS macro. The indirect effects were estimated using 5,000 bootstrap resamples, and the 95% confidence interval (CI) was based on bias-corrected estimates. A mediating effect was considered significant at *p* < 0.05 if the 95% CI did not include zero.

## 3 Results

### 3.1 Demographic characteristic and mental health state of participants

A total of 1,053 participants completed this investigation. Six cases were excluded due to obvious information errors, such as answering age questions with a name. As a result, 1,047 people were included in the final analyses, aged 17–75 years (31.47 ± 12.503 years). There were 328 (31.3%) men and 719 (68.7%) women. All participants were part of the medical system; most were staff (62.9%), and the remaining 37.1% were medical students. The education level for most participants was bachelor’s or higher (97.4%). For the original family structure, approximately 91% of participants reported living with their mother and father, as opposed to parental separation or single-parent rearing (Table 1).

**TABLE 1:** Demography characteristic mental health states of participants.

Variables	*n*	%	Variables	*n*	%
Gender			The only child		
Female	719	68.7	No	723	69.1
Male	328	31.3	Yes	324	30.9
Education background			Household income		
Junior college	98	9.4	Not good	43	4.1
Undergraduate	674	64.4	Not very good	90	8.6
Postgraduate	114	10.9	Average	750	71.6
Doctoral student	134	12.8	Good	146	13.9
Else	27	2.6	Very good	18	1.7
Permanent address			Original family		
Rural	225	21.5	Nuclear family	953	91.0
County	290	27.7	Blended family	49	4.7
Urban	532	50.8	Single-parent family	45	4.3
Living with parents during 0–3 year-old			Living with others during quarantine		
No	241	23.0	No	110	10.5
Yes	806	77.0	Yes	937	91.5
Profession			Adult attachment		
Health care workers	659	62.9	Secure	697	66.6
Students	388	37.1	Insecure	350	33.4

AAS, adult attachment style.

The results showed that 697 (66.6%) participants were securely attached, while 350 (33.4%) participants were insecurely attached. Approximately 24.9% of participants experienced physical discomfort, 8.5% experienced sleep problems, and 33.3% experienced negative mood (Tables 1, 2).

**TABLE 2:** Mental health state of participants.

Variables	*n*	Mean ± SD	Min	Max	Abnormal proportion (%)
Age	1,047	31.47 ± 12.503	17	75	
Perceived level of stress	1,047	5.19 ± 2.462	0	10	46.3
Mental health states	1,047	7.88 ± 4.8	0	25	
Physical discomfort	1,047	3.36 ± 2.823	0	10	24.9
Sleep problem	1,047	2.17 ± 2.206	0	9	8.5
Negative mood	1,047	4.21 ± 2.643	0	10	33.3
**Social support**					
Objective support	1,047	10.88 ± 3.694	3	67	
Subjective support	1,047	22.50 ± 4.979	11	32	
Utilization of support	1,047	7.76 ± 1.839	3	12	

SSRS, Social Support Rate Scale; CS, the current state of mental health. Abnormal proportion (%) for perceived level of stress, sleep scores, mood scores, and new physical discomfort: ≥6 are considered as abnormal population.

### 3.2 Pearson’s correlation analyses

The pairwise correlation analysis revealed significant positive relationships between the adult attachment styles and the three subtypes of social support and negative relationships with mental health states. Specifically, utilization support and subjective support were found to be significantly negatively related to mental health states. However, the correlation between objective support and mental health states was not significant. The statistical significance level was set at *p* < 0.05 (Table 3, Bonferroni correction).

**TABLE 3:** Pearson’s correlation analyses.

	Adult attachment	Utilization of support	Subjective support	Objective support	Mental health states
Adult attachment	1				
Utilization of support	0.222**	1			
Subjective support	0.403**	0.302**	1		
Objective support	0.253**	0.232**	0.400**	1	
Mental health states	−0.237**	−0.200**	−0.248**	−0.093	1

**p < 0.05 (two-tailed).

### 3.3 Further analyses

To determine whether age and gender are related to the variable, we used Pearson correlation. The Pearson correlation between age and gender with mental health status was not significant (*r* = 0.176, *p* = 0.935). However, there was a significant correlation between age and the dimensions of social support, including subjective support and objective support (*r* = 0.144, *p* < 0.001; *r* = 0.416, *p* < 0.001), as well as a significant correlation between gender and support utilization (*r* = −0.066, *p* = 0.033). Furthermore, when testing the correlation between adult attachment and mental health states while controlling for age and gender, there was a partial correlation (*r* = −0.257, *p* < 0.001). Similarly, when controlling for age and gender, the correlation between attachment and social support, including subjective support, objective support, and support utilization, remained significant, showing partial correlations (*r* = 0.227, *p* < 0.001; *r* = 0.337, *p* < 0.001; *r* = 0.224, *p* < 0.001). When controlling for age and gender, the correlation between the three dimensions of social support and mental health, including subjective support, objective support, and support utilization, also showed partial correlations (*r* = −0.100, *p* < 0.001; *r* = −0.292, *p* < 0.001; *r* = −0.202, *p* < 0.001). These findings indicate that although there are significant associations between age, gender, and social support, these associations remain significant when controlling for age and gender.

### 3.4 Mediation analysis

**FIGURE 2:**
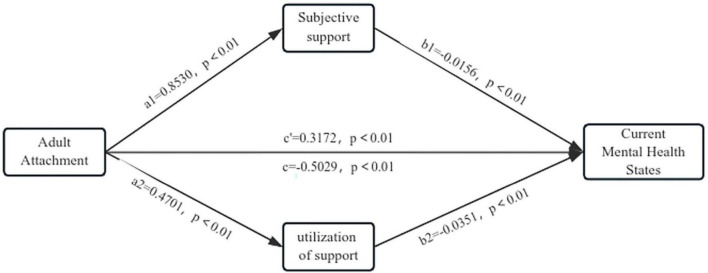
Correlation between adult attachment and mental health states mediated by subjective support and utilization of support. a, b, c, and c′ are standardized path coefficients.

As the correlation between objective support and mental health states was not significant, we used the adult attachment styles as the independent variable, subjective support and utilization of support as mediating variables, and mental health states scores as the dependent variable to conduct a mediation analysis. The results showed that adult attachment styles both directly and indirectly influenced mental health states (c′ = −0.3172, 95% CI: −2.1683 to −0.8770, c = −0.5 to 29, 95% CI: −3.0136 to −1.814) (Table 4). Subjective support and utilization of support play mediating roles in the relationship between attachment style and mental health states, respectively (ab_1_ = −0.1287, 95% CI: −0.9120 to −0.3341, ab_2_ = 0.0570, 95% CI: −0.4635 to −0.1132). Specifically, secure adult attachment positively predicted the subjective social support and utilization of social support (*a*_1_ = 0.8530, *p* < 0.01, *a_2_* = 0.4701, *p* < 0.01), while the latter negatively predicted mental health states (*b*_1_ = −0.0156, *p* < 0.001, *b_2_* = −0.0351, *p* < 0.01). The model is shown in Figure 2.

**TABLE 4:** Mediation effect analysis.

	Standardized coefficients	BootSE	95% CI		(ab/c)
			**LLCI**	**ULCI**	
Adult attachment → subjective support → mental health states	−0.1287	0.1468	−0.9120	−0.3341	0.2559
Adult attachment → utilization of support → mental health states	−0.0570	0.0885	−0.4635	−0.1132	0.1133
Direct effect	−0.3172	0.3290	−2.1683	−0.8770	0.6307
Total effect	−0.5029	0.3056	−3.0136	−1.8143	

The mediation effect size is ab/c.

## 4 Discussion

The relationship between attachment styles and mental health in medical staff and students during the COVID-19 pandemic has been largely unexplored. This study aimed to investigate the role of social support as a mediator between attachment styles (measured by Adult Attachment Scales) and the mental health states in this specific population during the COVID-19 quarantine (severe restrictions to social contact and the strict lockdown). A mediation analysis revealed that attachment styles had both direct and indirect effects on mental health state during quarantine. Specifically, our findings suggest that social support mediate the relationship between attachment styles and mental health state. This is consistent with previous studies that have shown similar mediation effects in different contexts, such as the association between COVID-19 and post-traumatic stress symptoms (Zeng et al., 2023), attachment avoidance and depressive symptoms in college students (Zhu et al., 2016), and chronic diseases and positive mental health (Yang and D’Arcy, 2022). In the current study, attachment style was chosen to be an independent variable, as it is thought to develop in early life and remain relatively stable (Fraley, 2019). The first critical path in the mediation model was that attachment styles significantly predicted mental health outcomes, which is consistent with previous research findings (Fearon et al., 2010; Flowers et al., 2018). Individuals with insecure attachment styles exhibited more negative affect, disturbed sleep, and physical symptoms (Fearon et al., 2010; Flowers et al., 2018). Another important path in the mediation model was the indirect effect of social support on the relationship between attachment styles and mental health. Although insecure attachment styles increased the risk of psychological disturbances, higher levels of social support could act as buffers, resulting in increased resiliency in response to stress. Subjective social support measures an individual’s evaluation of the support they receive from others, including feelings of care, understanding, and acceptance. Support utilization measures whether individuals actively seek and use support resources from others when facing difficulties. Therefore, although secure attachment cannot change objective social support, it can enhance individuals’ perception of support and encourage them to seek help and use available resources, ultimately promoting their mental health.

The results also showed that 33.3% of the participants reported emotional issues, 8.5% had sleep problems and 24.9% had physical discomfort. The prevalence of negative mood was comparable to previous studies among frontline workers; however, sleep problems seemed relatively mild, and physical discomfort was severer (Busch et al., 2021). This difference may be attributed to the different stages of the pandemic. In this study, the quarantine period occurred at the end of the epidemic, the disease had been well understood and the prognosis of the pandemic was relatively positive. Although medical personnel also faced quarantine and were on the front line, the risk had declined.

Furthermore, the findings indicated that attachment styles played direct and indirect roles in the mental health states during stress. Secure attachment resulted in better mental health both directly and indirectly. Social support mediated the relationship between adult attachment and mental health. This suggests that the positive effect of secure attachment on mental health was partly due to individual being able to get more social support. The correlation analysis revealed significant pairwise correlations between attachment styles, subjective social support, utilization of social support and mental health states, except for the correlation between objective social support and mental health states. Specifically, participants who reported insecure attachment exhibited lower levels of social support, worse mood, physical condition, and sleep quality. In other words, increased subjective support and utilization of support were associated with fewer mental health problems.

This finding was consistent with several previous studies that explored the relationship between social support and attachment in the general population (Pfaltz et al., 2022) and patients with physical pain (Charbonneau-Lefebvre et al., 2022). Securely attached individuals were more likely to recall and discuss painful experiences competently, feel satisfied and committed in their relationships, and experience psychological wellbeing (Gillath et al., 2016; Mikulincer and Shaver, 2016). In contrast, insecure or uncertain relationships can lead to self-doubt and various interpersonal difficulties. Social support involves mutual support between individuals; consequently, individuals with secure attachments tended to perceive more social support during the COVID-19 pandemic quarantine. Furthermore, social support provides access to resources that help individuals cope with difficulties during stressful situations (Fraley, 2019), ultimately improving stress responses (Daimer et al., 2022; Tsuno et al., 2022). This style aligns with our finding that increased social support was associated with fewer mental health problems and less perceived stress.

### 4.1 Theoretical and practical significance

It progresses to elucidate the influence of attachment styles on mental health states in the aftermath of stress. While several studies have explored the link between attachment and psychological wellbeing, this research examined the mediation role of social support. It may help inform decisions regarding support offered to individuals with insecure attachment, which in turn can at least partially help them reduce psychological distress (Adar et al., 2022). These findings may contribute to future research on the prevention and intervention of psychological problems after stress.

### 4.2 Strengths and limitations

Our study has several limitations that should be acknowledged. First, it is important to note that this study is cross-sectional and observational in nature. Future longitudinal studies would be beneficial in exploring the long-term effects of attachment styles on mental health. Second, all data in this study were obtained through self-report scales. Participants’ answers may be influenced by various factors, such as recall bias. It is important to consider these limitations when interpreting the results, as self-report measures may not always accurately reflect individuals’ actual experiences or behaviors (Maxwell and Cole, 2007). Future research could consider incorporating objective measures or multiple sources of data to enhance the validity of the findings. Additionally, it is worth noting that this investigation was conducted exclusively among medical staff and students in a large tertiary general hospital. Therefore, the generalizability of the results to other populations should be approached with caution (Dzakadzie and Quansah, 2023). Additionally, while all subjects experienced the same quarantine, it is important to acknowledge that the pandemic may have various other stressful factors, such as increased frequency of deaths, loss of relatives, working in non-professional fields, etc. Future research should strive to incorporate the relevant information to enhance the generalizability of the conclusions.

## Data availability statement

Due to the confidentiality of patients’ personal information, the authors can only provide partial information for data analysis which supporting the conclusions of this article. Requests to access the datasets should be directed to the corresponding author.

## Ethics statement

The studies involving humans were approved by the First Affiliated Hospital of Chongqing Medical University. The studies were conducted in accordance with the local legislation and institutional requirements. The participants provided their online consent to participate in this study.

## Author contributions

LD: Conceptualization, Funding acquisition, Supervision, Writing – original draft, Writing – review & editing. YY: Data curation, Formal analysis, Investigation, Methodology, Writing – original draft. KC: Data curation, Investigation, Methodology, Writing – original draft. KL: Formal analysis, Writing – original draft. WD: Supervision, Writing – review & editing. JG: Supervision, Writing – review & editing.

## Footnotes

1
https://www.wjx.cn/
